# Pilomyxoid astrocytoma of the corpus callosum presenting with primary haemorrhage in an adolescent

**DOI:** 10.1259/bjrcr.20150020

**Published:** 2017-01-07

**Authors:** Maria Longo, Juliano Adams Perez, Francine Oliveira, Apio Antunes, Leonardo Vedolin, Juliana Avila Duarte

**Affiliations:** ^1^Radiology, HMV, POA, Brazil; ^2^Radiology, HCPA, POA, Brazil; ^3^Pathology, HCPA, POA, Brazil; ^4^Neurosurgery, HCPA, POA, Brazil; ^5^Magnetic Resonance Unit, Hospital de Clinicas de Porto Alegre, Porto Alegre, Brazil

## Abstract

A 17-year-old male patient with history of intraventricular haemorrhage in 2007 underwent a brain MRI scan in 2013 owing to headache. Brain MRI scan showed an expansive lesion adjacent to the left lateral ventricle infiltrating the anterior portion of the corpus callosum. After surgery, pathology confirmed a pilomyxoid astrocytoma (PMA), an aggressive subtype of astrocytoma that occurs predominantly in the hypothalamic-chiasmatic region. On imaging, PMA presents as a tumour isointense on *T*_1_, hyperintense on *T*_2_ that enhanced heterogeneously with contrast. The *T*_2_ signal is higher than pilocytic astrocytoma, which indicates the presence of myxoid matrix. These findings on MRI scan have a direct correlation with a specific pathological finding—monomorphic proliferation of piloid cells in a mucopolysaccharide-rich matrix. These characteristics associated with the absence of Rosenthal fibres or eosinophilic granules indicated the diagnosis of PMA. To our knowledge, this is the first case report of PMA affecting the corpus callosum in an adolescent.

## Introduction

In November 2006, an international Working Group of 25 pathologists and geneticists met to suggest a new resolution for the brain tumours classification—the 2007 World Health Organization Classification of Tumours of the Central Nervous System. The goal of this new classification was the same of the older ones—grading the brain neoplasms. Based on six pathological criteria, these experts had the difficult task of classifying the tumours predicting theirs biological behaviour. The pathological criteria defined were the number of mitosis, presence of necrosis, vascular endothelial proliferation, nuclear polymorphism, cellular density and presence of genetic markers.^1^

The largest category between the primary neoplasms is the neuroepithelial tissue tumours, which encompass the astrocytomas. Anaplastic astrocytoma and glioblastoma are the most aggressive ones, classified as grades III and IV, respectively. The diffusely infiltrating astrocytomas are designated as grade II. One of the most benign-behaviours astrocytomas are the pilocytic one (grade I). However, there is a more aggressive subtype of pilocytic astrocytoma (PA), named pilomyxoid, classified as grade II.^1,2^

The pilomyxoid astrocytoma (PMA) occurs predominantly in the hypothalamic-chiasmatic region. To our knowledge, there is no previous case report that described a PMA affecting the corpus callosum (CC). Here, we describe the clinicopathological and radiological features of this patient with new observations.

## Clinical summary

An 11-year-old male, previously healthy, was brought to the emergency room with occipital headache and vomiting in 2007. He performed a brain CT scan, which showed a hyperdense area into the lateral left ventricle. A brain MRI scan performed in the same period confirmed a haemorrhagic lesion without abnormal enhancement adjacent to the CC. No vascular malformation was revealed by the conventional angiography. As the cause of the haemorrhage was not diagnosed, follow-up studies revealed that haematoma reduced in size and the clinical status of the patient improved, conservative management was chosen.

The neurosurgery team followed the patient until 2013, when owing to new intense headaches episodes, a new MRI scan was requested. The exam showed an expansive lesion in the left lateral ventricle infiltrating the anterior portion of the corpus callosum. The patient was submitted to a craniotomy followed by resection of the lesion. The details of the brain MRI scan and the histopathological findings are described in the foolowing section.

## Pathological and radiological findings

### Brain MRI scan

**Figure 1.**
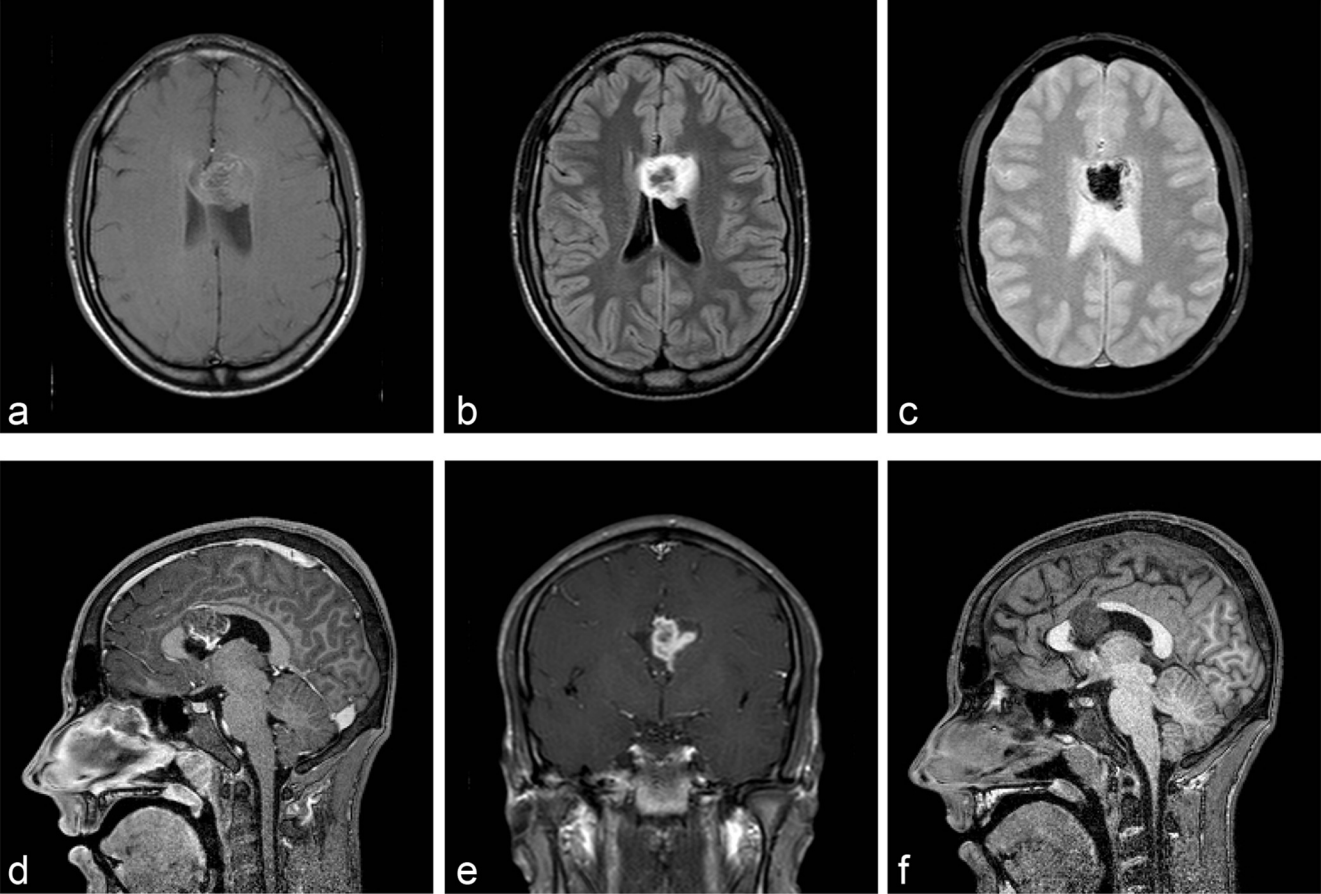
Axial brain MRI scan shows an intraventricular expansive lesion with hypointense signal on *T*_1_ (a) and hyperintense signal on FLAIR (b). Axial gradient echo shows the haemorrhagic component (c). Sagittal contrast-enhanced *T*_1_ weighted shows the lesion enhanced heterogeneously by the gadolinium (d). It occupies predominantly the anterior horn of the left lateral ventricle—coronal contrast-enhanced *T*_1_ (e) and infiltrates the anterior portion of the corpus callosum—sagittal *T*_1_ (f). FLAIR, fluid-attenuated inversion-recovery.

The brain MRI scan showed a solid lesion with cystic compound intraventricular expansive lesion affecting the anterior portion of the CC body that was hypointense on *T*_1_ and hyperintense on *T*_2_/fluid-attenuated inversion-recovery. It enhanced heterogeneously after gadolinium injection, presented a haemorrhagic component with blooming artefact that was hypointense on *T*_2_ gradient echo sequence and infiltrated the anterior horn of the left lateral ventricle predominantly (Figure 1).

## Pathological findings

The pathological study was performed on paraffin-embedded sections of the tumour. An immunohistochemical study was already performed for glial fibrillary acidic protein and Ki-67.

**Figure 2.**
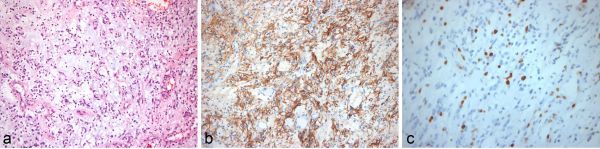
H&E (200×): the image shows a moderate cellularity neoplasm, consisting of rounded and oval cells without significant atypia, embedded in a myxoid stroma (a). GFAP (200×): strong positivity to GFAP, confirming the glial phenotype and showing cells with elongated processes (b). Ki-67: several cells stained with ki-67, demonstrating moderate proliferative index, beyond that expected for pilocytic astrocytoma and compatible with the diagnosis ofpilomyxoid astrocytoma (c). GFAP, glial fibrillary acidic protein; H&E, hematoxylin & eosin.

The hematoxylin & eosin stain showed a moderately cellularity neoplasm in a rich myxoid background. The cells were rounded and oval without significant atypia, and there was a discrete predominance around the vascular structures (Figure 2a). The glial fibrillary acidic protein study was strongly positive and showed elongated processes, which confirmed the glial origin of the tumour (Figure 2b). Moreover, in the ki-67 analysis, some cells were stained, showing a moderate proliferative index (Figure 2c). No Rosenthal fibers or eosinophilic granular bodies were found.

## Discussion

The PMA is a more aggressive subtype of PA, classified as World Health Organization grade II tumour.^1,2^ Both PMA and PA occur during the childhood although the PMA affects younger children and has a second peak of incidence in young adults. The neurological symptoms are unspecific, including headache and intracranial pressure increased. The pathological characteristic of PMA is the monomorphic proliferation of piloid cells in a mucopolysaccharide-rich matrix, without Rosenthal fibers or eosinophilic granules, commonly seen in PA.^3,4^

Traditionally, the PMA is found as a large mass in the hypothalamus/optic chiasm, with an H format. Most of the cases (60%) are suprasellar, with the geometric center in hypothalamus. However, owing to its expansive characteristic, the PMA generally invades the adjacent structures, such as deep gray nuclei, temporal lobes and closer white matter. The others 40% are located outside of the diencephalon, usually in the temporal lobe. Less commonly, it may occur in the midbrain, cerebellum, fourth ventricle and spinal cord.^5^ We are unaware of other cases originating in the corpus callosum.^6–11^ Typically, the presentation on the MRI scan is a hypointense imaging on *T*_1_ and hyperintense on *T*_2_/fluid-attenuated inversion-recovery sequences. In about half of the cases, the gadolinium enhancement is strong and heterogeneous.^3,5^

In our case, the PMA diagnosis was established in a 17-year-old patient, with a history of previous haemorrhagic vascular event.

The diagnosis was initially obscured by the extent of intraventricular haemorrhage. It was initially considered a vascular lesion or arteriovenous malformation as the most likely cause of the haemorrhage

Almost 25% of the cases of PMA bleed,^5^ which rarely occur in the PA.^5,13,14^ The tumour had a heterogeneous gadolinium enhancement, a pattern that is usually not found in PA as most of these tumours present a solid-cystic lesion, with a large cystic component.^14,15^ However, a heterogeneous enhancement usually suggest a glial tumour of a higher grade than lower grade gliomas.^3,5,13–15^

The final diagnostic was based on pathological findings. The main characteristics were the matrix predominantly myxoid and the proliferative index beyond that expected for PA. The absence of Rosenthal fibers and eosinophilic granular bodies, common in PA, ratified our hypothesis.^3,16^

The differential diagnostic between PA and PMA is not simple but owing to prognostic issues it should be done.^17^ Some imaging features such as haemorrhagic component, mainly solid mass, leptomeningeal dissemination and a higher *T*_2_ signal intensity inside the lesion suggest PMA (Table 1). These findings should alert the paediatric neuroradiologist to this specific diagnosis. Moreover, the neuropathologist should be alert to this hypothesis, to make the differential diagnosis actively, once the differences could be subtle.

**Table 1.** Differential diagnostic between PMA and PA^3,5,13–15^

	PMA	PA
Clinical features		
Age	Mean 18 months (second peak in young adults)	Mean 58 months
Symptoms	Signs of increased intracranial pressure	Signs of increased intracranial pressure and visual loss (pathway lesions)
Prognosis	Higher recurrence rate than PA	Survival rates at 20 years of 70%
Radiological feature		
Haemorrhage	Common (25%)	Rare (less than 8%)
T1WI	Large mass uniformly (60%) hypointense	Solid-cystic mass (solid portion is iso/hypointense)
T2WI	Hyperintense (homogeneous in 70%)	Hyperintense
Diffusion	Typically does not restrict	Same diffusivity of grey matter
Enhancement	Strong and heterogeneous enhancement	Strong and heterogeneous enhancement
Leptomeningeal metastases	Common	Rare
Location	Suprasellar (60%)	Cerebellum (60%) > Optic nerve/chiasm (30%)
Pathological features		
WHO grade	II	I
Proliferative index	Moderate	Lower to moderate
Myxoid stroma	Predominant	Poor
Calcification	Rare	Common
Rosenthal fibers	Absent	Present
Eosinophilic granular bodies	Absent	Present
Tumour cells	Monomorphic	Bipolar
Angiocentric patter	Frequent	Rare

PA, pilocytic astrocytoma; PMA, pilomyxoid astrocytoma; T1WI, *T*_1_ weighted image; T2WI, *T*_2_ weighted image; WHO, World Health Organization.

## Learning points

1. Despite PMA affecting the corpus callosum is a rare entity, it should be remembered in the differential diagnosis of lesions in this location;
2. Haemorrhagic component, mainly solid mass, leptomeningeal dissemination and a higher *T*_2_ signal intensity inside the lesion suggest PMA.
3. The differential diagnosis is with vascular lesion or arteriovenous malformation.

## Consent

Written informed consent for the case to be published (incl. images, case history and data) was obtained from the patient(s) for publication of this case report, including accompanying images.
